# Different Perspectives on Retest Effects in the Context of Spatial Thinking: Interplay of Behavioral Performance, Cognitive Processing, and Cognitive Workload

**DOI:** 10.3390/jintelligence11040066

**Published:** 2023-03-29

**Authors:** Benedict C. O. F. Fehringer

**Affiliations:** Department of Psychology of Education, University of Mannheim, 68131 Mannheim, Germany; benedict.fehringer@uni-mannheim.de

**Keywords:** retest effects, R-Cube-Vis Test, cognitive workload, viewing pattern, spatial thinking

## Abstract

Retest effects refer to performance improvements in a final test by completing previous tests with the same or similar testing materials. Improvements in test-related skills and/or increasing familiarity with the stimulus materials are considered sources of the retest effect. The present study analyzes retest effects in the context of spatial thinking, considering complementing perspectives (behavioral performance, cognitive processing, and cognitive workload). N = 141 participants completed a recently developed ability test for the visualization factor of spatial thinking (R-Cube-Vis Test). This test provides the opportunity to monitor the progression of changes in solving behavior from item to item within each of the six distinct difficulty levels. Items of one difficulty level all require the same spatial solving strategy but vary in visual appearance. Multi-level models were estimated, with items on level 1 and participants on level 2. Results demonstrated retest effects as changes from the beginning to the end of a set of items within each difficulty level by increasing accuracy. Gaze patterns showed the development of solving strategies by participants through, e.g., shifting the focus on relevant item parts. Increasing familiarity with the stimulus materials was indicated in reduced reaction times and increased confidence ratings, but also by the results of a pupillary-based cognitive workload measure. Furthermore, differences between participants with overall high vs. low spatial ability were considered. In addition to a deeper understanding of the underlying mechanisms of the retest effect, the complementing perspectives provide more detailed information about individual ability profiles for diagnostic purposes.

## 1. Introduction

The goal of the present work is to provide insights into the underlying mechanisms of the retest effect. The retest effect is defined as an increasing test score “after prior exposure to an identical test or to an alternate form of this test under standardized conditions” (S. 982, Lievens et al. 2005). The effect is robust and can be found in various settings, such as ability testing under controlled conditions (see meta-analyses of Hausknecht et al. 2007; Scharfen et al. 2018) as well as in real-world classrooms (see meta-analysis of Schwieren et al. 2017).

Studies that analyze the underlying mechanisms of the retest effect vary in focus. Some studies consider complete test scores; for example, Lievens et al. (2007) proposed the presence and absence of a measurement bias, as well as predictive bias, as indicative of different explanations of the retest effect. Other studies focus on information per item to observe the cognitive processing steps during test performance, e.g., by considering reaction times per item (Heil and Jansen-Osmann 2008) and self-reports (Glück and Fitting 2003).

The present study aims at exploring the retest effect in ability testing in the domain of spatial thinking. In this domain, ability tests commonly use a series of similar items to obtain a test score (e.g., the paper folding test PFT, Ekstrom et al. 1976a, the mental rotation test MRT, Vandenberg and Kuse 1978). In the domain of spatial thinking, explanations of the retest effect have focused on memory effects (e.g., Carpenter and Pashler 2007; Heil et al. 1998; Tarr and Pinker 1989). Changes in cognitive strategies to solve similar items that may underly the retest effect have not been systematically explored yet.

The focus of the present study is the investigation of changes in cognitive processing from item to item for an explanation of the retest effect in ability testing in the domain of spatial thinking. To this end, not only accuracy measures and reaction times are used, but more fine-grained techniques of eye tracking and pupillometric analyses are also applied. 

The application of eye tracking and pupillometric techniques for the analyses of changes in cognitive processing from item to item is only possible with suitable visual test materials. The items of commonly used tests, such as the PFT or the MRT, are too heterogenous, differ in difficulty as well as in visual appearance and, thus, hamper the interpretation of gaze patterns. Therefore, a recently developed and validated test for spatial thinking is used in the present study. This test, the R-Cube-Vis Test for spatial thinking (Fehringer 2020b), was developed for use with eye tracking specifically. Moreover, the test conforms to the linear logistic test model, consisting of six distinct difficulty levels with average accuracy values from nearly perfect (.99) to nearly chance level (.53) for the long version (Fehringer 2020b). At each level, the items are homogeneous and equivalent. They can be solved following one rational strategy, although the items differ in visual appearance. Therefore, the test allows analysis of cognitive processes in each difficulty level in more detail by observing and comparing changes in gaze patterns. The present study utilizes the R-Cube-Vis Test to monitor the development of solving behavior within a test (i.e., by solving a series of equivalent test items), and to gain insights into the retest effect from item to item within the difficulty levels of the test. In addition, accuracy measures and reaction times, as well as pupillometric measures indicating cognitive load, are taken into consideration. 

### 1.1. The Retest Effect

There are three commonly proposed explanations of the retest effect, i.e., the gain of test scores from the first test to the retest (e.g., Arendasy and Sommer 2017; Lievens et al. 2007; Scharfen et al. 2018). (1) There is an actual improvement in the measured ability; (2) there is an improvement of test-related skills (such as applying solving strategies and item memorization) that are not related to the construct; or (3) participants are growing familiar with the stimulus materials. However, equal underlying mechanisms might be assigned to different explanations, depending on the context. For example, memorizing specific item solutions might be construct-related, e.g., for learning word pairs (Arnold and McDermott 2013; Pyc and Rawson 2010) but construct-unrelated if the test demands the participants to manipulate a certain object (Heil et al. 1998; Tarr and Pinker 1989).

According to explanation 1, the retest effect occurs if participants improve their ability in the measured construct. Hence, the results of the retest are as valid as the results of the first test (Lievens et al. 2007).

The second explanation for the retest effect (explanation 2) addresses the improvement of skills to optimize the solving behavior of the specific test. Participants might adapt existing strategies or develop new solving strategies for the items of cognitive ability tests (e.g., Hayes et al. 2015; Verguts and Boeck 2002). Hence, these solving strategies are specific for the particular testing materials to some extent. For example, participants may learn from test to test (or from item to item) to focus on the relevant parts of the item figures of this particular test, as was demonstrated for Raven’s matrices (e.g., Hayes et al. 2015; Verguts and Boeck 2002). This is also in accordance with results from studies comparing experts and novices; experts outperform novices in visuospatial tasks by focusing on relevant parts and ignoring irrelevant parts (e.g., Charness et al. 2001; Jarodzka et al. 2010; van Gog et al. 2005). Such results can also be obtained if participants were trained (to “experts”) during the experiment (e.g., Canham and Hegarty 2010; Hegarty et al. 2010). However, these observations apply to repeated attempts to solve items of the same or an alternative form of the test. Although these improved skills are related to the construct, an improvement of ability, in the sense of transfer to another test measuring the same construct (e.g., spatial thinking) or to more general facets of intelligence, is not addressed here, i.e., there is usually no gain in general intelligence (Hayes et al. 2015). Additional to improving solving strategies, explanation 2 also encompasses item memorization in cognitive ability tests (Arendasy and Sommer 2017). The gain of test scores in the retest is a result of memorization of item solutions from the first test. Therefore, the validity of the retest is reduced if only test-related skills are improved (Lievens et al. 2007).

If, however, the learning goal is memorization of, e.g., word pairs, then item memorization, which is called the “testing effect”, can be assigned to explanation 1. The testing effect refers to the mnemonic benefits from repeated retrieval (retesting) of the learning materials from memory (Roediger and Butler 2011), as compared with studying (learning) the materials again. The testing effect has been studied extensively with verbal declarative knowledge as learning materials. It can be found for word pairs, e.g., for Russian–English (Arnold and McDermott 2013) or Swahili–English (Pyc and Rawson 2010); similar free-recall and paired-associate learning tasks (see Roediger and Karpicke 2006); but also for daily quizzes (e.g., in an introductory psychology classroom, Batsell et al. 2017; Wooldridge et al. 2014); and the studying of scientific texts for non-university populations (Meyer and Logan 2013). The testing effect occurs independently of the participant’s cognitive ability (Jonsson et al. 2020). Although in some studies the testing effects also refer to higher-level effects, such as transfer knowledge (Jensen et al. 2020), in the present study the testing effect is specifically interpreted as a memory retrieval effect, whereas the retest effect generally refers to a gain in test scores independent of the underlying mechanisms.

The third explanation for the retest effect (explanation 3) addresses familiarity effects with the testing material (Anastasi 1981). Participants who do not know the test itself and the utilized materials must get familiar with the stimuli and the specific tasks. Test scores achieved in this phase might, therefore, underestimate their ability in the measured construct. The more familiar the participants get with the test, the more the test scores might increase during this familiarization phase. This familiarization phase is usually shorter or even not existing in the retest, which should lead to higher and more valid test scores in the retest (Lievens et al. 2007).

### 1.2. Retest Effects in the Context of Spatial Thinking

Spatial thinking is an important intelligence factor (Paivio 2014) and builds a significant basis for ability development in STEM (science, technology, engineering, mathematics) domains (Wai et al. 2009). According to the factor-analytic approach, spatial thinking can be divided into two to six factors, depending on the underlying construct definition (see Hegarty and Waller 2005). For the present study, the focus will be on the main factor of spatial thinking, visualization, based on the definition of the extensive work of Carroll (1993), who surveyed and reanalyzed 94 datasets. In his definition, he refers to Ekstrom et al. (1976b), who defined visualization as “the ability to manipulate or transform the image of spatial patterns into other arrangements” (Ekstrom et al. 1976b, p. 173). According to the more recent 2 × 2 classification of Uttal et al. (2013), visualization belongs to tasks demanding intrinsic and dynamic information (in contrast to extrinsic and static information). Intrinsic information is needed to manipulate the presented object itself, and dynamic information is needed to mentally rotate or manipulate (parts of) this object.

Retest effects in the context of spatial thinking occur as retrieval effects with identical stimulus materials (e.g., Carpenter and Pashler 2007; Heil et al. 1998; Tarr and Pinker 1989), but also as effects for transfer tasks in the final test (e.g., Carpenter and Kelly 2012; Rohrer et al. 2010). For example, retrieval effects were found in a visuospatial task (Carpenter and Pashler 2007), in which participants had to remember features of maps and mentally place them back into partially blank maps. Repeated testing showed higher performance in the final test than repeated studying of the map. Additionally, mental rotation tasks could be solved faster in final tests if the same tasks were tested before (Heil et al. 1998; Tarr and Pinker 1989). However, such memory-based effects in the domain of spatial thinking do not lead to construct-related gains in test scores, but show test-specific improvements through item memorization (explanation 2).

Retest effects that occur for transfer tasks in the final test might indicate improvements in construct-related ability that are necessary to perform the final test (according to explanation 1) but also to the improvement of test-specific skills, such as solving strategies for the specific tasks (explanation 2). For example, Rohrer et al. (2010) let participants assign regions and cities to locations on maps, and could show that participants who were previously tested on the material benefited if previous tests showed the same maps but with easier tasks. In another study by Carpenter and Kelly (2012), participants first had to perform pointing tasks in a certain environment by imagining standing at one object, looking at a second object, and pointing at a third object. There were three conditions (guided pointing, test with feedback, and test without feedback). The final pointing test used the same environment, from the first phase, containing known tasks, but also new pointing tasks. The authors found, in the final test, that participants of the test condition (with and without feedback) outperformed participants who were guided to the correct answer in the first test. Both studies suggest an improvement of general test-related skills, over and above memorizing previously performed tasks. However, it could not be determined which specific changes occurred during testing and how the participants solved the items in the final test.

An important aspect of the retest effect that occurs in transfer tasks in the final test is the development and usage of solving strategies. It is well known that participants differ in their solution strategies if they perform spatial thinking tests. These strategies are distinguishable between efficacy and effectivity (e.g., Glück and Fitting 2003; Heil and Jansen-Osmann 2008; Mayer and Massa 2003). Some studies found that different strategies lead to comparable accuracy and might only differ in reaction times (Glück and Fitting 2003; Heil and Jansen-Osmann 2008). Other studies could show that specific strategies are related to higher performance (Bethell-Fox et al. 1984; Nazareth et al. 2019), and, therefore, are crucial to gaining higher test scores. Explorative results based on eye tracking techniques (Fehringer 2020b) showed differences in the viewing patterns between participants with higher and lower overall performance in the R-Cube-Vis Test for spatial visualization. Participants with higher performance focused more on that part of the items that contains the relevant information to solve this item. Furthermore, participants with higher performance showed a more systematic viewing pattern for difficult items and a less systematic pattern for medium items. This last result is in accordance with the results of Nazareth et al. (2019), who found an advantage to a switching pattern compared to a fixating pattern while solving the mental rotation task (based on Shepard and Metzler 1971).

In addition to solving strategies that are indicated by gaze movements, pupillometric measures might also provide insight into the retest effect by revealing changes in cognitive load during a series of tests. Pupillary responses are related to cognitive workload (c.f. Beatty 1982; Kahneman 1973), but also to changes in light (Steinhauer et al. 2004) and fatigue (Andreassi 2007). A promising pupillometric-based measure that controls for influences of light and fatigue is the Index of Pupillary Activity (IPA, Duchowski et al. 2018), which was also optimized for application with the R-Cube-Vis Test (Fehringer 2020a). A study with the same materials could show that the IPA can indicate an increasing cognitive workload from the easiest levels to the most difficult levels of the R-Cube-Vis Test (Fehringer 2021a). Since the IPA is sensitive to changes in cognitive workload, the IPA might also be able to indicate changes in cognitive workload from the first test item to the last test item within the same difficulty level. As a result of higher familiarity with the stimulus materials, the cognitive workload is expected to decrease. Such an observation during testing would be consistent with explanation 3 of the retest effect (familiarity). However, this might only be true if participants were able to solve the items. If the tasks are too difficult, pupil dilation can decrease, indicating less cognitive workload (e.g., Granholm et al. 1996; van der Meer et al. 2010). This was also found by Zekveld and Kramer (2014) who analyzed pupil reactions during tasks of masked speech. Participants with a lower ability reported giving up more often in the most difficult conditions, compared to participants with a higher ability. Consequently, the pupil reactions of participants with a lower ability indicated less cognitive workload for more demanding tasks. Therefore, if participants were not able to solve the items, a decreasing cognitive workload would not be informative regarding familiarity, but might indicate that participants would not attempt to solve the items. Therefore, it is necessary to study pupillometric data, intended to indicate cognitive workload, together with other measures such as actual accuracy, reaction times, and solving strategies. For example, if participants just guess the answers, they should be less accurate and faster.

To the best knowledge of the author, there are no studies that systematically analyze the development of solving strategies as a possible explanation for the retest effect in the domain of spatial thinking. The present study aims to monitor (nearly) continuous changes in solving behavior during testing by minimizing the intervals between single tests. According to the results of Carpenter and Kelly (2012) as well as Carpenter and Pashler (2007), it is expected that changes can be detected within short intervals (the intervals in both studies were 10 and 30 min, respectively). To this end, the R-Cube-Vis Test (Fehringer 2020b) was conducted by interpreting each item as a single “test”, exploiting the conformity of the R-Cube-Vis Test to the linear logistic test model. Therefore, systematic changes indicate the development of solving behavior from item to item, since the items within each difficulty level are interchangeable regarding the demanded cognitive processes. In this way, the R-Cube-Vis Test can reveal progressions in solving behavior from the first to the last item within each of its six difficulty levels.

### 1.3. Summary and Hypotheses

The present study analyses the retest effect in the domain of spatial thinking using the R-Cube-Vis Test (Fehringer 2020b), concerning the potential explanations: improvement of test-related skills (explanation 2) and familiarity with the test materials (explanation 3). Retest effects according to explanation 1 (improvement of construct-related ability) are not expected. The time window to develop one’s spatial thinking ability is quite short (only a single test run), and, even with longer time windows, gain in scores of ability tests usually indicates no gain in general ability (Hayes et al. 2015). Furthermore, it is not expected that items can be solved by item memorization, since all test items differ pairwise and are only presented once. Hence, knowledge about the solution of one item is not transferable to another item.

In contrast to standard tests, the R-Cube-Vis Test has specific characteristics designed to gather more detailed information from different perspectives. In addition to accuracy and reaction times, the R-Cube-Vis Test also asks for a confidence rating about the given answer for each item. Furthermore, the R-Cube-Vis Test is specially developed and validated for the usage of eye tracking. Due to its homogenous difficulty levels, which conform to the linear logistic test model, and the simple structured items, the R-Cube-Vis Test allows the monitoring of continuous changes of cognitive processing steps from item to item. The three different perspectives addressed—behavioral perspective (accuracy, reaction times, confidence ratings), cognitive processing perspective (eye movement measures), and cognitive workload perspective (pupillometric measure)—allow for a more detailed analysis of the solving behavior and connecting different information for more accurate interpretations.

Based on the existing literature, a retest effect is expected from the first item to the last item, i.e., there will be an increase in accuracy. According to explanation 2, eye tracking data will show that participants will improve their solving strategies by shifting their focus from irrelevant information to relevant information during each of the six difficulty levels from the first to the last item, i.e., fixations should be more on relevant parts, fixation patterns should be more systematic at the end, and fixation sequences should be more similar. According to explanation 3, participants will be more familiar with the testing materials at the end than at the beginning, i.e., participants will be more confident and faster at the end. Moreover, pupillometric analyses will indicate decreasing cognitive workload. However, it is taken into consideration that these general patterns are confounded with a tendency to give up, especially for participants with a lower ability at the most difficult levels. The expected accuracy rates in the two most difficult levels are expected to be around .75 and .53, respectively, over all participants (according to Fehringer 2020b) with a chance level of .50. Participants with a lower ability should have even lower accuracy rates at these difficulty levels. Therefore, the specific hypotheses are differentiated between the difficulty levels of the R-Cube-Vis Test (i.e., between easy, medium, and difficult levels). In addition to the item position as the first independent variable, the test performance as an indicator of spatial thinking ability is considered the second independent variable (see Fehringer 2020b). All hypotheses are formulated for each considered measure of one of the three perspectives and are summarized in Table 1.

**Behavioral perspective, performance in each item (“item accuracy”).** It is expected that retest effects will be found within each difficulty level, i.e., participants will increase their performance from the first item to the last item (Hypothesis 1a). Trivially, participants with higher test performance (higher ability) should also have higher item accuracy (Hypothesis 1b).

**Behavioral perspective, reaction times.** It is expected that participants will grow more familiar with the stimulus materials (explanation 3) and, therefore, they will become faster from the beginning to the end within each level (Hypothesis 2a). Participants with a higher ability will be faster, but only in the easy levels. It is expected that participants with a lower ability will tend to give up in the most difficult levels and would, therefore, show lower reaction times (Hypothesis 2b).

**Behavioral perspective, confidence rating.** It is expected that participants will grow more confident in their answers from the first to the last item, i.e., more familiar with the testing materials, within each difficulty level according to explanation 3 (Hypothesis 3a). Furthermore, it was expected that participants with a higher ability would generally be more confident in their answers (Hypothesis 3b).

**Figure 1:**
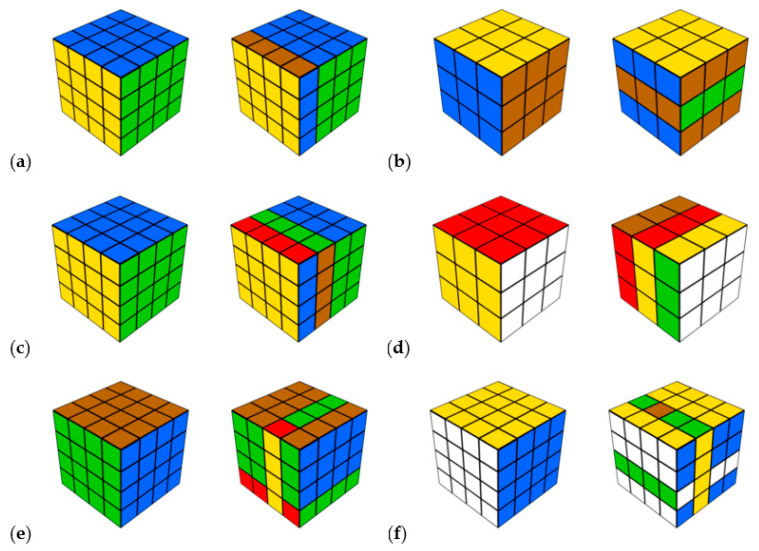
Possible items of the R-Cube-Vis Test for each difficulty level, ordered from easy (**a**) to difficult (**f**). The task is always to decide whether both cubes are equal, except for rotated elements.

**Cognitive processing perspective, gaze fixations on relevant parts.** It is expected that participants will increase their gaze fixations on relevant parts of the items in all difficulty levels, according to explanation 2 (Hypothesis 4a). In particular, according to a previous study (Fehringer 2020b), it is expected that participants will fixate more on the right cube in the two easiest and two most difficult levels and fixate relatively more on the left cube in both medium levels (Level c and d, Figure 1). According to Fehringer (2020b), the preference for relevant parts will also be found in participants with a higher ability, especially for the more difficult levels (Hypothesis 4b). For easy and medium items, the effects could only descriptively be found in Fehringer (2020b).

**Cognitive processing perspective, systematic viewing pattern.** It is expected that participants will increasingly show a systematic viewing pattern from the beginning to the end, according to explanation 2 (Hypothesis 5a). Akin to gaze fixations on relevant item parts, participants with a higher ability will show more systematic viewing patterns, especially in the most difficult levels. However, according to Fehringer (2020b), the reversed result is expected for the medium levels (Hypothesis 5b). The tasks in the medium levels demand more gaze switching between the two presented cubes than in the other difficulty levels (Figure 1, in Materials). In the two easiest and two most difficult levels, the original version of the cube (left cube) can be easily derived from the right cube. In both medium levels, the original version is harder to detect.

**Cognitive processing perspective, similar viewing patterns from item to item.** It is expected that participants learn during each difficulty level how to optimally solve the items (explanation 2). Therefore, the viewing pattern of two consecutive items will be more similar at the end than at the beginning of each level (Hypothesis 6a). Participants with a higher ability will generally show more similar viewing patterns than participants with a lower ability (Hypothesis 6b).

**Cognitive workload perspective.** It is expected that the cognitive workload decreases from the beginning to the end within each difficulty level as the participants get more familiar with items (explanation 3), but also find solving strategies according to explanation 2 (Hypothesis 7a). Furthermore, similar results as for reaction times are expected regarding the participants’ ability. In the easiest levels, participants with a higher ability will have a lower cognitive workload than participants with a lower ability. This pattern will be reversed for the most difficult levels due to the tendency of participants with a lower ability to give up (Hypothesis 7b).

## 2. Method

### 2.1. Participants

The study was conducted with N = 177 participants who were all from a large German university. They were not color-blind and received course credit for participation. Thirty participants were discarded due to invalid eye tracking data. Another three participants had test performance lower than the chance level (50%) and a further three participants stated under comments that they did not perform the study seriously. The final sample consists of N = 141 participants, 111 female and 30 male, with an average age of M = 21.61 years (SD = 2.80 years) ranging from 17 to 34 years.

### 2.2. Materials

The long version of the R-Cube-Vis Test (Fehringer 2020b) was conducted with 288 items from six difficulty levels. Each level consists of 48 items (24 possible and 24 impossible). Each item shows two Rubik’s cubes. The cube on the left side is always solved, whereas the cube on the right side has one or two rotated elements (Figure 1). The items between different levels differ regarding the size of the cubes (3 × 3 × 3 vs. 4 × 4 × 4), the number of rotated items (one vs. two), and how the elements are rotated (parallel vs. crossed). The items within each level differ regarding the color combination of the six colors, the axis around which rotated, and the rotation direction. Participants always have to decide whether the left cube can be maintained by back-rotating the rotated elements of the right cube (possible items) or not (impossible items). A correct answer is counted with 1 and an incorrect answer with 0. The final score is the accuracy value over all possible items, i.e., the number of correctly solved items divided by 144.

After each item, participants had to state their confidence from 1 (very sure) to 4 (very unsure). All six levels are presented in a fixed order of increasing difficulty (according to Fehringer 2020b), from a to f (Figure 1). Before each level, four trial items were presented. The 48 items within each block were randomly presented. The R-Cube-Vis Test is a spatial thinking test and validated for the visualization factor (Fehringer 2020b, 2021b). The correlation with the paper folding test as a specific visualization test is r = .51 and the reliability estimate is α = .90 for the long version of the R-Cube-Vis Test (Fehringer 2020b). In the current study, the reliability estimate is α = .95. The R-Cube-Vis Test conforms to the linear logistic test model with its six difficulty levels. Furthermore, the participants had to indicate their sex, age, and study major.

### 2.3. Procedure

After a short introduction, the R-Cube-Vis Test was administered via a computer using an eye tracker. Before every single level, the eye tracker was recalibrated, due to the long duration of the test and the possible deterioration of the calibration (Holmqvist et al. 2011). Finally, participants had to provide information about their sex, age, and study major. The complete session took a maximum of 90 min.

### 2.4. Apparatus

The used eye tracker was the Tobii TX300 (recording rate: 300 Hz) embedded in an eye tracker unit with a screen (screen size: 23’’, aspect ratio: 16:9, resolution: 1920 × 1080 pixels). It was connected to the presentation software E-Prime 2.0 (Psychology Software Tools Inc. 2012) with the “Extensions for Tobii” (Psychology Software Tools Inc. 2011). The average distance between the participant’s eyes and the eye tracker was M = 62.09 cm (SD = 5.48 cm). The participants were calibrated using the nine-point calibration procedure of the presentation software. The goodness of the calibration was visually evaluated by comparing the estimated gazes with the expected fixations. If these estimations were out of the indicated tolerance range, the calibration procedure was repeated.

### 2.5. Data Preparation

All eye tracking data labeled as invalid by E-Prime (validity value: 4, Psychology Software Tools Inc. 2012) were discarded, as well as all values falling outside the screen limits. The remaining estimated gaze points were assigned to fixations, saccades, and glissades using the adaptive algorithm presented by Nyström and Holmqvist (2010) with the optimization proposed by Fehringer (2018). The algorithm assigns each data point to one of these three events. All remaining data points were labeled as undefined/noise. The proportion of this undefined data per participant was M = .09 (SD = .06). The considered pupillometric measure was the Index of Pupillary Activity (IPA, Duchowski et al. 2018), which is an indicator of cognitive workload and controls for the influence of light and fatigue. The algorithm was optimized by Fehringer (2020a). In the present study, the z-standardized values of the difference between both eyes are considered because they showed the strongest effects between the difficulty levels (Fehringer 2020a).

### 2.6. Analyses

According to the formulated hypotheses, the study analyzed the influence of item position as the first independent variable, and test performance in the R-Cube-Vis Test (indicator for spatial thinking ability) as the second independent variable. Due to the nested data structure (each participant completed each item), one multi-level model (MLM) was computed, with items on level 1 and participants on level 2, for each hypothesis, i.e., for each dependent variable. Since it was expected that the changes from the first to the last items differ between the six difficulty levels within a single dependent variable (see the differentiated hypotheses), each level for each dependent variable was considered separately in the first step. The final general model for each hypothesis was maintained in a second step including all levels.

The procedure was the same for each dependent variable. In the first step, seven MLMs were computed for each difficulty level, i.e., 42 MLMs altogether. The seven models differ regarding the included independent variables (item position, test performance) and whether random slopes were considered. Model 1 is the constant model, including a random intercept. Models 2a and 2b added item position as the first independent variable. Models 3a and 3b included test performance as the second independent variable. Model 4a and 4b further extended Model 3a and 3b by including the interaction term between item position and test performance. All a-models (Model 2a, 3a, 4a) had only a random intercept, whereas all b-models additionally included a random slope. Afterward, the models were compared with each other to find the best-fitting model according to the information criteria AIC and BIC. The model with the lowest AIC and BIC values was selected.

In the second step, all selected models, one for each difficulty level, were analyzed in a single MLM. However, to avoid a shrinking effect and due to the different specifications of the models, the six difficulty levels were included with six dummy variables (with coding 1 for the referred level and 0 for all other levels). This allows for defining specific intercepts, main effects, and interaction effects for each level separately, with the general intercept set to 0 (Gelman and Hill 2006).

The two independent variables are test performance, ranging from 0.5 to 1, and item position, which is standardized with a uniform distribution on the interval from 0 (first possible item) to 1 (last possible item). The three dependent variables of the *behavioral perspective* are item accuracy (values 0 or 1, Hypothesis 1a/b), logarithmized reaction times (>0, Hypothesis 2a/b), and confidence rating (from 1 to 4, Hypothesis 3a/b). The confidence rating is a subjective measure. Participants had to rate their confidence in their given answer after each item. The confidence rating scale goes from 1 (very sure) to 4 (very unsure). The MLM with the binary dependent variable item accuracy was estimated with the binomial distribution. The basic measures for the hypotheses of the *cognitive processing perspective* are fixations on one of the whole cubes. Fixations on the cubes are robust measures over all items within each difficulty level, and they are indicative of correctly solved items. More fixations on the right cube are related to higher item accuracy for the two easiest and two most difficult levels, but they are related to lower item accuracy for the two medium levels (Fehringer 2020b). Therefore, the relative number of fixations on the left cube was included as a dependent variable, with values from 0 to 1 for Hypothesis 4a/b. The degree of systematic viewing patterns (Hypothesis 5a/b) is indicated by the entropy (Shannon 1948). The entropy is a measure of uncertainty, and delivers values > 0. Smaller values indicate a more systematic viewing pattern. The similarity between two fixation patterns of consecutive items (Hypothesis 6a/b) is measured by the Levenshtein distance ratio. The Levenshtein distance (Levenshtein 1966) compares two strings (in the present study, two fixation sequences) and computes the minimal number of transformation steps to transform one string into the other one. The fewer transformations are needed, the more similar the two strings are. To control for different fixation lengths, the Levenshtein distance was standardized. The final Levenshtein distance ratio (LDR) ranges from 0 to 1, whereby larger values indicate higher similarity between two fixation sequences. Hypothesis 7a/b (*cognitive workload perspective*) is tested using the z-standardized difference value between both eyes of the IPA.

### 2.7. Transparency and Openness

Research materials are available at Disciplinary Repository for Psychological Science, PsychArchives, Fehringer (2021c). Data were analyzed using R, version 4.1.2 (R Core Team 2021) with the packages psych, version 2.1.9 (Revelle 2021), lme4, version 27.1 (Bates et al. 2015), lmerTest, version 3.3.5 (Kuznetsova et al. 2017), plyr, version 1.8.6 (Wickham 2011), and ggplot2, version 3.3.5 (Wickham 2009). Data and syntax are available as Supplementary Materials. The study design as well as the analysis were not preregistered.

## 3. Results

First, the data were cleaned from corrupted recordings, implausible reaction times, and erroneous eye tracking data (see above). The descriptive data are presented in Table 2.

All hypotheses were tested with the estimated MLMs described above with a separate MLM for each dependent variable. The independent variable test performance refers to the sum score of the complete test (as the measure of the participant’s ability).

**Figure 2:**
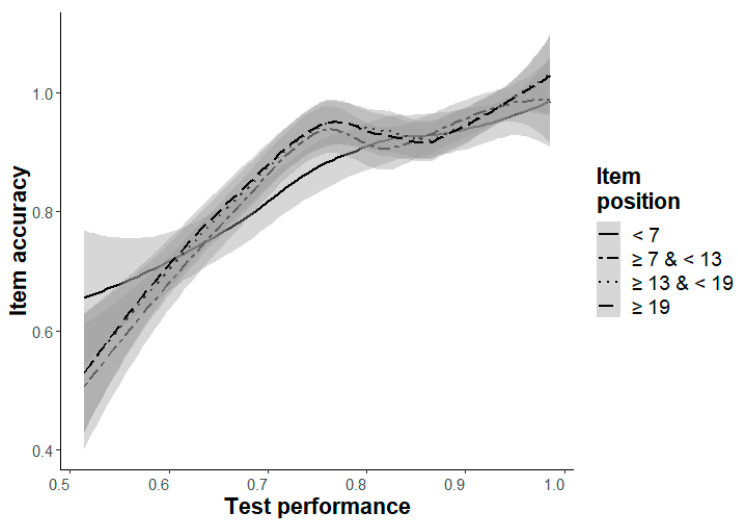
Changes of item accuracy depending on test performance (ACC-poss, x-axis) and item position (grouped into four intervals, indicated by four lines) with a .95-confidence-interval for Level a.

**Item accuracy (Hypothesis 1a/b).** The results confirmed the expected retest effect (Table 3). The item accuracy increases from the first to last item in each difficulty level (Hypothesis 1a, except for Level a), with a significant main effect for the independent variable item position, b ≥ 0.45, *p* ≤ .006. The unexpected negative effect in Level a can be explained by the included interaction effect between item position and test performance, which can be traced back to the items at the beginning (Figure 2). If the same model is estimated without this interaction effect (i.e., with Model 3b instead of 4b for Level a), the expected retest effect can also be found as a main effect of item position in Level a, b = 1.79, *p* < .001. Trivially, there are significant main effects of test performance on item accuracy for each difficulty level (Hypothesis 1b), b ≥ 6.01, *p* < .001.

**Reaction times (Hypothesis 2a/b).** The results show the expected pattern with increasing reaction times from the beginning to the end in each difficulty level, b ≤ −0.10, *p* ≤ .002 (Hypothesis 2a, Table 4). The expected differential pattern for the test performance as the independent variable (Hypothesis 2b) could also be found, with faster reaction times for participants with a higher ability and slower reaction times for participants with a lower ability, in the two easiest levels (Levels a and b), b ≤ −0.65, *p* < .001. The opposite result was found for the two most difficult levels (Levels e and f), b ≥ 1.02, *p* < .001 (Table 4).

**Figure 3:**
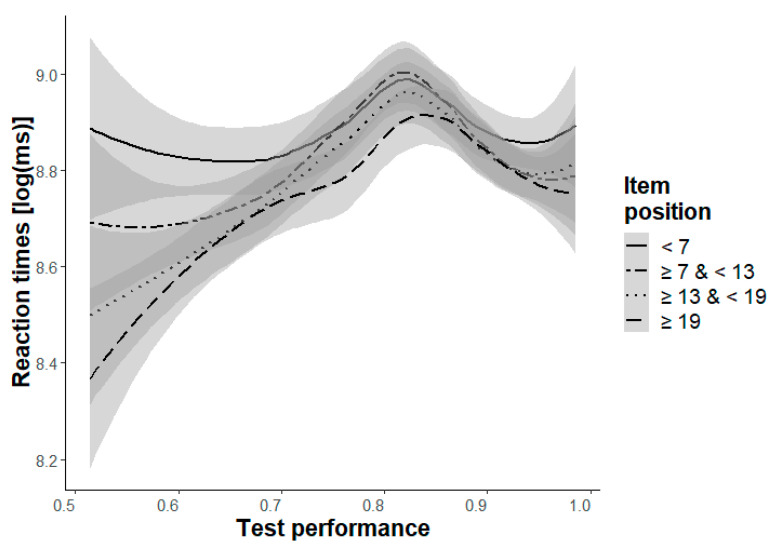
Changes of the logarithmized reaction times depending on test performance (ACC-poss, x-axis) and item position (grouped into four intervals, indicated by four lines) with a .95-confidence-interval for Level d.

Remarkably, Model 2b had the best fit for the medium levels (Levels c and d), which includes item position as the only independent variable, not test performance. The inspection of the plot for Level d (Figure 3) reveals a bell-shaped curve, which might suggest that participants with a lower ability tended to guess the items and, therefore, had faster reaction times, whereas participants with a higher ability tried to solve the items seriously and, therefore, had slower reaction times.

**Figure 4:**
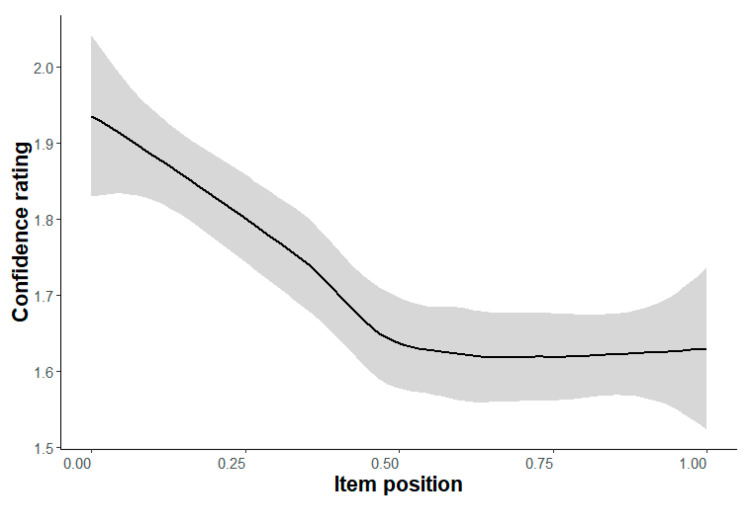
Changes of the confidence rating depending on item position with .95-confidence-interval for Level a.

**Confidence Rating (Hypotheses 3a/b).** The confidence rating (ranging from 1, high confidence, to 4, low confidence) changed only in the easiest level (Level a) from the beginning (lower confidence) to the end (higher confidence), b = −0.33, *p* < .001 (Table 5). Remarkably, the negative slope appeared in the first half of the items exclusively; in the second half of the items, there was a flat line (Figure 4). This might indicate that participants get more familiar with the kind of items in the first half of the first difficulty level, until they reach their “real” confidence level. Participants might also be familiar with the following levels since the items of these levels use the same stimulus material (except for the size) with similar manipulations (one or two parallel rotations). In Level e, however, there was also a tendency of the participants to be more confident about their answer at the end than at the beginning, b = −0.11, *p* = .057 (Table 5), which might indicate that participants are (more) unfamiliar with this type of items than of the preceding difficulty levels. The reason might be that the items show cubes with cross-rotated elements instead of parallel rotations of elements as in Levels a to d. Therefore, Hypothesis 3a could only be partially confirmed. The expected main effect of test performance on confidence rating (Hypothesis 3b) could be found for all difficulty levels (except for Level f) with b ≤ -0.94, *p* ≤ .011 (Table 5), i.e., participants are more confident about their answers if they have a higher ability.

**Fixations on the left cube (Hypothesis 4a/b).** As formulated in Hypothesis 4a, there is a significant decrease in the relative number of fixations on the left cube from the beginning to the end in the easiest levels (Levels a and b) and Level e, b ≤ −.03, *p* < .001 (Table 6). In Level f, however, there was no significant main effect of item position. As expected, the opposite pattern with an increasing relative number of fixations on the left cube could be observed in the medium levels (Levels c and d), b ≥ .03, *p* < .001. Hypothesis 4b could be confirmed for the most difficult levels (Levels e and f), with relatively fewer fixations on the left cube for participants with a higher ability compared to participants with a lower ability, b ≤ −.30, *p* < .001 (Table 6).

**Entropy (Hypothesis 5a/b).** It was expected that participants would show more systematic viewing patterns at the end than at the beginning within each difficulty level, i.e., a decreasing entropy was expected. However, this pattern could only be found in the two easiest levels as a significant effect in Level a (b = −.05, *p* < .001) and descriptively in Level b (Table 7). For the levels c, d, and f, the pattern is inverse, b ≥ .04, *p* ≤ .002. As expected in Hypothesis 5b, there was a significant increase in entropy (indicating less systematic viewing behavior) for participants with a higher ability compared to participants with a lower ability in the most difficult levels (Levels e and f), b ≤ −.42, *p* < .001. The reversed pattern, however, only occurs in the easiest levels (Levels a and b), b ≥ .16, *p* ≤ .010, with significantly decreasing entropy values. Notice, the results are similar to reaction times.

**Figure 5:**
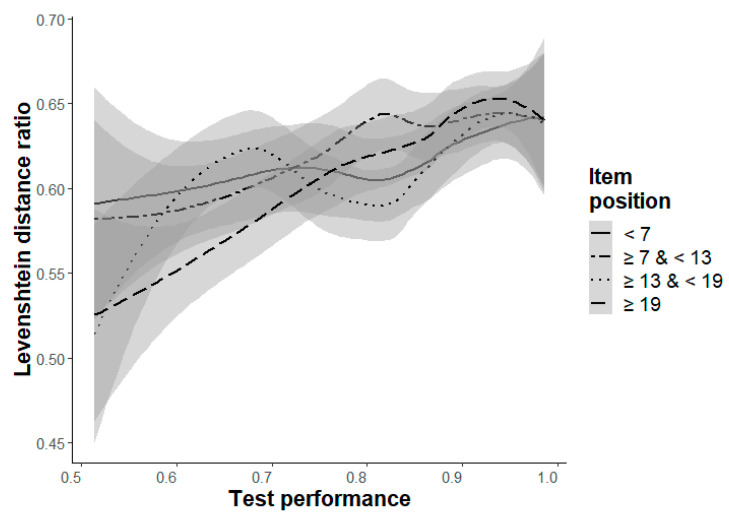
Interaction effect of item position (grouped in four intervals, indicated by four lines) with .95-confidence-interval and test performance (ACC-poss, x-axis) on the Levenshtein distance ratio for Level d.

**Levenshtein distance ratio (LDR, Hypothesis 6a/b):** Hypothesis 6a could not be confirmed. The only significant main effect was a decreasing LDR (i.e., decreasing similarity) from the beginning to the end in Level d, b = −.21, *p* = .005 (Table 8). However, the significant interaction effect, b = .24, *p* = .006, indicates a dependence on participants’ test performance. Figure 5 shows that the LDR only decreases for participants with a lower ability, and descriptively shows the expected pattern for participants with medium and higher abilities. The main effect of test performance with a higher LDR for participants with a higher ability (Hypothesis 6b) could only be found for the two most difficult levels, b ≥ .17, *p* < .006 (Table 8). In the other levels (except for Level b), there was, however, a trend in the expected direction.

**Figure 6:**
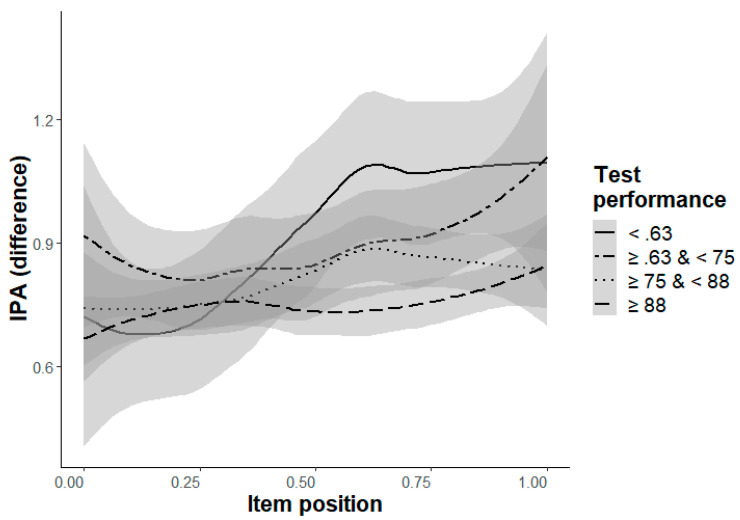
Changes of IPA (difference) depending on the item position (x-axis) and the test performance (ACC-poss, grouped in four intervals, indicated by four lines) with .95-confidence-interval for Level e. The four test performance groups consisted of N = 11 (ACC-poss < .63), N = 23 (≥.63 and <.75), N = 44 (≥.75 and <.88), N = 63 (≥.88) participants. Notice, larger values of IPA (difference) indicate lower cognitive workload, and the item position is standardized with the range 0 (1st item) to 1 (24th item).

**Cognitive workload, difference of IPA values (Hypothesis 7a/b).** A significant decrease in cognitive workload from the beginning to the end (Hypothesis 7a) could be found for the two easiest levels (Levels a and b) and for Level e, b ≥ .16, *p* ≤ .006, as well as descriptively for Level f, b = .06, *p* = .183 (Table 9). Remarkably, this pattern could also be found for the confidence rating (Table 5) with the largest effects in Levels a and e. Please note that in both levels new transformation aspects are demanded, a simple rotation of elements (Level a) and crossed rotation of elements (Level e). As formulated in Hypothesis 7b, participants with a higher ability showed lower cognitive workload in the easiest levels (Level a and b), b ≥ .56, *p* < .001. In the most difficult level (Level f), participants with a higher ability showed higher cognitive workload, but only descriptively b = −.92, *p* = .148 (Table 9). However, the effect is significant if the interaction effect is included, b = −.87, *p* = .003, as well as if the MLM only considers Level f (without the other levels), b = −.86, *p* < .001. The significant interaction effect in Level e is due to the unexpected distribution of the IPA in the lowest ability participants’ group (accuracy < .63, Figure 6). All participants with higher test performance (accuracy ≥ .63) show the expected ordering, with higher cognitive workload (lower IPA values) for participants with a higher ability than for participants with a lower ability (Figure 6). Please note, the distribution of Level f is similar to the expected ordering over all participants with test performance ≥ .63.

## 4. Discussion

The present study utilized the R-Cube-Vis Test for the investigation of retest effects in spatial thinking. Different measures were considered to investigate the retest effect from complementing perspectives (behavioral, cognitive processing, cognitive workload), regarding improvements in construct-related ability and increasing familiarity. Changes from the beginning to the end of a set of items within each difficulty level of the R-Cube-Vis Test were analyzed. The overall sum score of participants as an indicator of spatial ability was considered.

The retest effect was observed within each difficulty level as increasing accuracy from the first to the last item. These results confirm that the retest effect can be found for short intervals in the domain of spatial thinking, consistent with results reported by Carpenter and Kelly (2012), and Carpenter and Pashler (2007), who examined time intervals of 10 and 30 min, respectively, in opposition to longer test–retest intervals (e.g., one day in the study of Rohrer et al. 2010).

One explanation for the retest effect is an improvement of test-related skills (explanation 2), such as applying appropriate solving strategies for the presented stimulus materials. To this end, three different measures based on gaze movement (fixation proportion, systematic viewing patterns, and similarity of viewing patterns) were analyzed in the present study. Comparisons between the proportion of fixations on the left and the right cube confirmed the shift during testing from (mainly) irrelevant item features to relevant item features. This is consistent with studies considering the retest effect (e.g., Hayes et al. 2015; Verguts and Boeck 2002). In the end, participants focus more on the right (twisted) cube in the easiest and most difficult levels since all information to solve the item is easily accessible from this cube. In the medium level, however, focus on the left cube is more supportive since the relevant information is more challenging to extract exclusively from the right cube (see also Fehringer 2020b). A strategic adaption of the solving strategy, that goes beyond a simple focus shift toward relevant parts, is thus observable. The results suggest that participants change their attention distribution to optimize their solving behavior, depending on the specific difficulty level and based on insights about the relevance of visual information for the solution.

Analyses of entropy supported this interpretation. Although the expected decreasing trend for entropy (as an indicator for unsystematic viewing patterns) was only (at least descriptively) observable in the easiest levels, there were significant unexpected changes in entropy in the medium levels, as well as the most difficult level. These significant changes showed increasing entropy. They still indicate that participants adapted their viewing behavior from the first to the last item within these levels. The reason why the pattern differed between difficulty levels might be a result of the “distribution” of the relevant visual parts which differed between the levels. Paralleling the finding regarding fixations, an increased entropy might indicate that participants adapted to the need of finding a new solution strategy at a new difficulty level, in which the relevant visual information had to be found on other parts of the shown objects. However, strategy changes associated with entropy should be analyzed in more detail in follow-up studies. The increasing similarity between consecutive items (measured by Levenshtein distance ratio) could only be found in Level d and only for participants with a higher ability. The found interaction effect at this level indicates that these changes differ between ability groups.

Effects on changes of these three cognitive processing measures, regarding inter-individual differences between participants in spatial ability, support the known effects between experts and novices (e.g., Hegarty et al. 2010; Jarodzka et al. 2010) in the most difficult levels. Participants with a higher ability focused more on relevant parts, showed more systematic viewing patterns, and had more similar fixation sequences than participants with a lower ability. These differences in similarity were also found descriptively for the second most difficult level and the medium levels. The opposite pattern in entropy could be found for the easiest levels (more unsystematic viewing behavior of participants with a higher ability) might be due to the simple items, since participants with high ability recognize the solution without applying specific solving strategies.

The retest effect can also be the result of increasing familiarity with the stimulus materials (explanation 3, Anastasi 1981). To this end, two behavioral measures (reaction times and confidence ratings) and the Index of Pupillary Activity (IPA, Duchowski et al. 2018), indicating cognitive workload, were considered. Reaction times showed the expected decreasing changes from the first to the last item in each difficulty level. Increasing confidence within each level was descriptively found in all levels, however, the increase was only (marginally) significant in the easiest and in the second most difficult level (Level a and e). The results of cognitive workload were similar; a significantly decreasing cognitive workload was found in Levels a and e. In addition, the workload decreased in the second easiest level (Level b). The reason might be that the stimulus materials in Level a and e are different compared to the items that the participants know at the beginning of these levels. Notably, items of Level a are the first items that the participants see at all. The items of Level b to d are similar to the items of Level a since all items of these four levels (Levels a to d) show cubes with “simple” rotated elements. Even if two elements are rotated (Levels c and d), each element, when considered on its own, looks familiar compared to the already seen rotated elements. New kinds of rotation, however, occur in Level e with cross-rotated elements. In opposite to the parallel rotated elements, there is a clear ordering of which element is rotated first and which is rotated second. Additionally, the two visible sides of the rotated element show more than two different colors. These special characteristics of Levels a and e are also reflected in the reaction times, with stronger effects than in Levels b, c, and d. Besides Levels a and e, Level f shows a familiarity effect, but only if the “objective” measures are considered, with a similarly strong effect in reaction times and marginal significant decreasing workload. The subjective confidence rating, however, indicates no familiarity effect in Level f. These results demonstrate a correspondence between the different measures, but also additional information that can only be revealed by the objective measures.

Stronger differences between the objective measures and the confidence rating could be observed when considering the participant’s ability. As expected, both objective measures show faster reaction times and less workload in the easiest levels (Levels a and b) for participants with a higher ability. No effects could be found in the medium levels (Levels c and d). As expected, slower reaction times and descriptively higher workload were found in the most difficult levels (Levels e and f) for participants with a higher ability. This pattern reflects the tendency of participants with a lower ability to give up if items became too difficult. The participants’ confidence ratings (subjective measure) show a unique pattern for all difficulty levels (except Level f with no effect), with generally higher confidence in the answers of participants with a higher ability, compared to participants with a lower ability.

Further correspondence is noticeable between entropy and reaction times. The different entropy patterns between participants with higher and lower abilities for the difficulty levels (higher entropy of participants with a higher ability in the easiest levels and lower entropy of participants with a higher ability in the most difficult levels) correspond to the results found for the reaction times, where participants with a lower ability show faster reaction times than participants with a higher ability in the most difficult levels, presumably because they tend to give up. An analogous explanation might apply to entropy. Participants with a higher ability might be less challenged in the easiest levels and, therefore, solve the items with minimal effort, and might, therefore, wander around with their gaze. When participants with a higher ability were challenged to show their full ability potential, they showed more systematic viewing behavior to solve the items, whereas the gazes of participants with a lower ability wandered unsystematically around and showed, correspondingly, more unsystematic patterns.

In summary, the results of the present study confirm the retest effect for the considered stimulus materials in the context of spatial thinking, and show that the retest effect is observable (as increasing accuracy) in small time intervals from item to item. The results of the considered measures from the three different perspectives support two explanations for the found retest effect, as improvements of test-specific skills (explanation 2) as well as increased familiarity with the stimulus materials (explanation 3). The improvements in skills are reflected by the development of appropriate solving strategies for the presented stimulus materials. The development of solving strategies is indicated by an increase in similar gaze movements, and a shift to more relevant item parts from the beginning to the end of the presented items within a specific difficulty level. Increasing familiarity within specific difficulty levels can be observed in faster reaction times from the beginning to the end. In addition, the subjective confidence rating and the pupillary-based cognitive workload measure reflect familiarity. However, the observed results for these indicators differ between the six difficulty levels, and depend on the participant’s ability.

Comparisons between the easiest and the most difficult levels show differences between the participants with lower and higher abilities. Participants with a higher ability seem to solve the easiest items without developing specific solving strategies, whereas participants with a lower ability might tend to give up if the items are too difficult in the most difficult levels. In both cases, this results in less structured gaze movements. In the case of participants with a lower ability, the tendency to give up is reflected in lower performance, and a correspondingly lower cognitive workload. Future work, however, could investigate the issue of low performance with higher difficulty more thoroughly. Participants may give up because they are indeed unable to solve the items, but it could also be that they gain lower test scores because they do not fully exploit their cognitive resources, as indicated by the low cognitive workload.

The study is limited to the used stimulus materials and the domain of spatial thinking. In particular, the results of the viewing behavior can only be transferred to other testing materials with difficulty. However, the general approach with complementing perspectives and measures could be adapted for other scenarios investigating retest effects. Although the more elaborative measures of entropy and Levenshtein distance delivered significant contributions, the partly unexpected results also show that alternative measures might be explored in future research to indicate the relevant cognitive processes.

To the best knowledge of the author, this is the first study that systematically investigates the underlying mechanisms of the retest effect from complementing perspectives in the domain of spatial thinking. The results provide insights into the interplay between participants’ behavior, the corresponding cognitive processes, and the cognitive workload during the solving of the items. Furthermore, the setting and the special properties of the R-Cube-Vis Test allow differentiation between objective and subjective measures, and fine-grained monitoring of the changes of these measures during the test. The study demonstrates the connection between these sources of information (e.g., a correspondence between the objectively measured workload and the subjective confidence rating) and lays the groundwork for future testing procedures in diagnostic contexts. If we not only know about the performance of a participant (by accuracy and reaction times), but additionally gather information about the applied solving strategies (by viewing pattern-based indicators) and invested cognitive workload (by pupillometric measures), we can draw significantly more sophisticated conclusions about the participants’ ability than is possible with current standard testing procedures.

## Figures and Tables

**Table 1:** Hypotheses for each independent variable and differentiated between difficulty levels.

Hypothesis	Begin → End (Item Position)	Low → High (Ability Indicated by Test Performance)
1a/b	more accurate	more accurate
2a/b	faster	faster (e) slower (d)
3a/b	more confident	more confident
4a/b	more fixations on relevant parts	more fixations on relevant parts (d)
5a/b	more systematic	less systematic (e) more systematic (d)
6a/b	more similar	more similar
7a/b	less cognitive workload	less cognitive workload (e) more cognitive workload (d)

Note. “e”: easier levels; “m”: medium levels; “d”: difficult levels.

**Table 2:** Descriptive results.

	M	SD	Range	Skewness	Kurtosis
Accuracy	.83	.38	.00 –1.00	−1.76	1.09
Logarithmized reaction times	8.62	0.64	6.23–10.98	−0.08	0.05
Confidence interval	1.89	0.97	1.00–4.00	0.79	−0.46
Relative number of fixations	.18	.16	.00–.83	0.55	−0.53
Entropy	.38	.17	.01–0.69	−0.24	−0.99
LDR	.63	.18	.04–1.00	−0.30	−0.34
IPA	0.94	0.82	0.00–6.79	1.61	3.68

**Table 3:** Fixed effects and correlations between random intercept (RI) and random slope (RS) of the MLM with the dependent variable item accuracy.

Difficulty Level	Model	Fixed Effects	b	se	*p*	r(RI, RS) ^c^
Level a	4b	Intercept	−2.77	0.95	.004	.10
		Item position	−1.97	1.91	.302	
		Test performance	6.41	1.23	<.001	
		Interaction ^a^	4.57	2.58	.076	
Level b	3b	Intercept	−3.08	0.75	<.001	.23
		Item position	1.77	0.51	<.001	
		Test performance	7.43	0.98	<.001	
Level c	3a ^b^	Intercept	−4.82	0.55	<.001	-
		Item position	0.50	0.18	.006	
		Test performance	8.26	0.68	<.001	
Level d	3a ^b^	Intercept	−5.08	0.64	<.001	-
		Item position	0.57	0.17	.001	
		Test performance	8.29	0.79	<.001	
Level e	3a ^b^	Intercept	−6.44	0.58	<.001	-
		Item position	0.64	0.16	<.001	
		Test performance	9.46	0.71	<.001	
Level f	3a ^b^	Intercept	−4.51	0.49	<.001	-
		Item position	0.45	0.13	<.001	
		Test performance	6.01	0.59	<.001	

Note: ^a^ Interaction between item position and test performance. ^b^ Model 3a contains no random slope. ^c^ “r(RI, RS)” refers to the correlation between random intercept and random slope.

**Table 4:** Fixed effects and correlations between random intercept (RI) and random slope (RS) of the MLM with the dependent variable reaction times.

Difficulty Level	Model	Fixed Effects	b	se	*p*	r(RI, RS)
Level a	3b	Intercept	9.31	.18	<.001	−.51
		Item position	−0.32	.03	<.001	
		Test performance	−0.96	.21	<.001	
Level b	3b	Intercept	8.64	.15	<.001	−.36
		Item position	−0.10	.03	.002	
		Test performance	−0.65	.18	<.001	
Level c	2b	Intercept	8.82	.03	<.001	−.43
		Item position	−0.11	.03	.002	
Level d	2b	Intercept	8.89	.03	<.001	−.29
		Item position	−0.13	.03	<.001	
Level e	3b	Intercept	8.15	.18	<.001	−.47
		Item position	−0.32	.04	<.001	
		Test performance	1.02	.21	<.001	
Level f	3b	Intercept	6.85	.22	<.001	−.40
		Item position	−0.34	.04	<.001	
		Test performance	2.63	.26	<.001	

**Table 5:** Fixed effects and correlations between random intercept (RI) and random slope (RS) of the MLM with the dependent variable confidence rating.

Difficulty Level	Model	Fixed Effects	b	se	*p*	r(RI, RS)
Level a	3b	Intercept	2.86	.34	<.001	−.49
		Item position	−0.33	.07	<.001	
		Test performance	−1.18	.41	.004	
Level b	3b	Intercept	2.26	.30	<.001	−.01
		Item position	0.04	.05	.397	
		Test performance	−0.94	.36	.011	
Level c	3b	Intercept	3.50	.32	<.001	−.49
		Item position	−0.04	.05	.432	
		Test performance	−1.98	.38	<.001	
Level d	3a	Intercept	3.21	.32	<.001	−
		Item position	−0.02	.04	.541	
		Test performance	−1.67	.38	<.001	
Level e	3b	Intercept	3.53	.27	<.001	−.30
		Item position	−0.11	.06	.057	
		Test performance	−1.73	.32	<.001	
Level f	2b	Intercept	2.49	.07	<.001	−.46
		Item position	−0.05	.07	.501	

**Table 6:** Fixed effects and correlations between random intercept (RI) and random slope (RS) of the MLM with the dependent variable relative number of fixations on the left cube.

Difficulty Level	Model	Fixed Effects	b	se	*p*	r(RI, RS)
Level a	2b	Intercept	.31	.01	<.001	−.58
		Item position	−.11	.01	<.001	
Level b	2b	Intercept	.23	.01	<.001	−.47
		Item position	−.06	.01	<.001	
Level c	2b	Intercept	.15	.01	<.001	−.33
		Item position	.03	.01	<.001	
Level d	2b	Intercept	.24	.01	<.001	−.47
		Item position	.04	.01	<.001	
Level e	3b	Intercept	.37	.03	<.001	.55
		Item position	−.03	.01	<.001	
		Test performance	−.31	.04	<.001	
Level f	3b	Intercept	.35	.04	<.001	−.12
		Item position	−.00	.01	.593	
		Test performance	−.30	.05	<.001	

**Table 7:** Fixed effects and correlations between random intercept (RI) and random slope (RS) of the MLM with the dependent variable entropy.

Difficulty Level	Model	Fixed Effects	b	se	*p*	r(RI, RS)
Level a	3b	Intercept	.28	.06	<.001	−.66
		Item position	−.05	.01	<.001	
		Test performance	.18	.07	.006	
Level b	3b	Intercept	.27	.05	<.001	−.45
		Item position	−.01	.01	.313	
		Test performance	.16	.06	.010	
Level c	2b	Intercept	.30	.01	<.001	−.54
		Item position	.05	.01	<.001	
Level d	2b	Intercept	.42	.01	<.001	−.56
		Item position	.04	.01	<.001	
Level e	3b	Intercept	.59	.05	<.001	−.42
		Item position	.00	.01	.986	
		Test performance	−.42	.06	<.001	
Level f	3b	Intercept	.63	.06	<.001	−.31
		Item position	.04	.01	.002	
		Test performance	−.50	.07	<.001	

**Table 8:** Fixed effects and correlations between random intercept (RI) and random slope (RS) of the MLM with the dependent variable Levenshtein distance ratio.

Difficulty Level	Model	Fixed Effects	b	se	*p*	r(RI, RS)
Level a	3b	Intercept	.52	.05	<.001	−.65
		Item position	−.01	.02	.732	
		Test performance	.11	.06	.065	
Level b	1	Intercept	.63	.01	<.001	−
Level c	3b	Intercept	.60	.04	<.001	
		Item position	−.01	.01	.366	
		Test performance	.06	.05	.234	
Level d	4a	Intercept	.61	.05	<.001	−
		Item position	−.21	.07	.005	
		Test performance	.02	.06	.685	
		Interaction	.24	.09	.006	
Level e	3b	Intercept	.40	.06	<.001	.17
		Item position	.00	.02	.834	
		Test performance	.29	.07	<.001	
Level f	3b	Intercept	.48	.06	<.001	.26
		Item position	.02	.02	.282	
		Test performance	.17	.07	.006	

**Table 9:** Fixed effects and correlations between random intercept (RI) and random slope (RS) of the MLM with the dependent variable IPA (difference between both eyes).

Difficulty Level	Model	Fixed Effects	b	se	*p*	r(RI, RS)
Level a	3a	Intercept	.50	.13	<.001	
		Item position	.26	.04	<.001	
		Test performance	.56	.15	<.001	
Level b	3a	Intercept	.55	.18	.004	
		Item position	.16	.05	<.001	
		Test performance	.76	.22	<.001	
Level c	1	Intercept	.87	.05	<.001	
Level d	1	Intercept	.81	.02	<.001	
Level e	4a	Intercept	.80	.19	<.001	
		Item position	.89	.32	.006	
		Test performance	−.09	.22	.681	
		Interaction	−.88	.39	.022	
Level f	3a	Intercept	1.57	.54	.004	
		Item position	.06	.05	.183	
		Test performance	−.92	.06	.148	

## Data Availability

The data presented in this study are available in supplementary material.

## Supplementary Materials

The following supporting information can be downloaded at: https://www.mdpi.com/article/10.3390/jintelligence11040066/s1.
